# Bridging Gaps in Sundown Syndrome Research: a Scoping Review and Roadmap for Future Multimodal Approaches

**DOI:** 10.1093/arclin/acaf062

**Published:** 2025-07-03

**Authors:** Qianru Xu, Feng Vankee Lin, Yang Liu, Guoying Zhao

**Affiliations:** CogT Lab, Department of Psychiatry and Behavioral Sciences, Stanford University, Stanford, CA, USA; Center for Machine Vision and Signal Analysis, University of Oulu, Oulu, Finland; CogT Lab, Department of Psychiatry and Behavioral Sciences, Stanford University, Stanford, CA, USA; CogT Lab, Department of Psychiatry and Behavioral Sciences, Stanford University, Stanford, CA, USA; Center for Machine Vision and Signal Analysis, University of Oulu, Oulu, Finland; Center for Machine Vision and Signal Analysis, University of Oulu, Oulu, Finland

**Keywords:** Sundown syndrome, Dementia, Behavioral disturbances, Multimodal approach, Caregiver support

## Abstract

**Objective:**

Sundown syndrome (SS), or sundowning, is a neuropsychiatric phenomenon marked by the worsening of symptoms in the late afternoon or evening, primarily in individuals with dementia. By systematically examining previous studies, this scoping review aims to (1) bridge traditional questionnaire-based assessment methods with advanced sensor-based tools and (2) propose a multimodal framework to guide future research in enhancing risk identification, diagnosis, monitoring, and treatment across key symptom categories.

**Method:**

We conducted a comprehensive review of Web of Science, PubMed, Medline, APA PsycInfo, and IEEE Xplore to identify studies on SS. Following established scoping review guidelines, 13 review papers and 41 empirical studies were selected and analyzed based on traditional questionnaire-based observation and/or sensor-based measurement methods.

**Results:**

We identified key limitations in traditional assessment methods and classified SS symptoms into five domains: psychomotor symptoms, cognitive and perceptual disturbances, mood and affective symptoms, psychosis, and disruptions in activities of daily living and instrumental activities of daily living. Building on these insights, we proposed a multimodal platform integrating sensor technologies to enhance risk identification, diagnosis, continuous monitoring, and treatment.

**Conclusions:**

This study advances the understanding of SS by synthesizing prior research, refining symptom domains, and proposing a roadmap for future investigation and intervention. The integration of multimodal sensor technologies holds the potential to reduce caregiver burden, enhance patient care, and enable more effective management of SS and other behavioral disturbances in older adults.

## INTRODUCTION

Sundown syndrome (SS), also known as sundowning, is characterized by a spectrum of cognitive, emotional, and behavioral changes that primarily occur during sunset or late afternoon. Despite being documented for decades (Cameron, 1941; Evans, 1987) and widely accepted by clinicians, the understanding of SS remains incomplete. For instance, some studies have failed to find evidence of SS (e.g., Bliwise et al., 1993; Harper et al., 2007). Researchers have suggested that this may be due to factors related to caregivers such as nurse fatigue and understaffing in nursing homes rather than patient-related factors (Bedrosian & Nelson, 2013). Moreover, there are still no universally accepted operational definitions of SS such as what kinds of symptoms and time ranges should be included, making it difficult to identify, diagnose, monitor, and provide appropriate and timely treatment for SS. However, despite these formal definitions and unresolved research questions, the distress of SS on both patients and caregivers is real. Many more people search for information on SS online during the winter months, with state-by-state data showing a significant decrease in searches as daily sunlight increases and a rise in searches with higher latitudes (Madden & Feldman, 2019). Furthermore, behavioral disturbances such as wandering, agitation, and physical aggression from patients present safety risks to both patients and their caregivers (Cipriani et al., 2015). These disruptive behaviors not only increase the likelihood of physical injury for patients but also significantly contribute to caregiver strain and mental health problems, such as depression and stress, thereby substantially increasing management costs (Desai et al., 2012; Schnaider Beeri et al., 2002). Considering the aforementioned aspects, it is crucial to understand and assess SS in a practical and, ideally, labor-saving manner, to assist patients and help in alleviating the burden on caregivers.

Nowadays, with the continuous advancement in artificial intelligence (AI) techniques and the integration of various sensors, such as cameras, smartphones, smartwatches/fitness bands, radars, Wi-Fi, and virtual reality/augmented reality (VR/AR) technologies, into our daily lives, it is becoming increasingly feasible to automatically detect and offer timely intervention/treatment for various conditions (for reviews, see Khan et al., 2024; Sujith et al., 2022). In the context of dementia, machine learning (ML) approaches have demonstrated their power in risk assessment, detection, diagnosis, and aiding in dementia care, as well as in forecasting patients’ prognoses (Lyall et al., 2023; Su et al., 2022). With these technological advancements and an understanding of SS’s timing-related characteristics, multimodal data recording and AI solutions open new possibilities for the comprehensive management of SS.

Though there are already some systemic or scoping reviews (e.g., Boronat et al., 2019; Cipriani et al., 2015; Khachiyants et al., 2011; Yevchak et al., 2012) that provided various insights into SS, including its prevalence, causes, and both pharmacological and non-pharmacological solutions in treatment (see Table 1), a notable gap remains. Specifically, there is a lack of studies that focused on the practical measurement of SS and provided guidance on integrating computer technology and neuroscience to study SS from an interdisciplinary perspective. Therefore, this scoping review seeks to thoroughly examine and assess the existing literature on SS with the following objectives: (1) to profile previous studies and summarize the characteristic and measurement methods, with an emphasis on linking traditional questionnaire-based observational approaches with advanced tools and sensors for measuring SS; (2) to develop a framework for using multimodal sensors (“Multimodal data recording platform”) to aid in risk identification, diagnosis, monitoring, and treatment of SS across different symptoms categories (i.e., Psychomotor Symptoms, Cognitive and Perceptual Disturbances, Mood and Affective Symptoms, Psychosis, and activities of daily living [ADLs]/instrumental activities of daily living [IADLs]), based on insight from previous studies and the Biomarkers, Endpoints, and other Tools (BEST) framework (see Box 1). By incorporating professional expertise with advanced measurement and analysis, we aim to provide a hybrid approach that benefits both patients and caregivers. This will contribute to the ongoing debates surrounding SS and inform evidence-based practices in geriatric care. Ultimately, we hope our research will guide future studies and aid in the development of tools to more effectively assist patients and their caregivers.

**Table 1:** Summary of the main content from previous review papers

Study	Definition	Symptoms and Characteristics	Pathological Analysis	Recommended Solutions	Main Content
(Duckett, 1993)	“A marked increase in confusion, disorientation and possibly agitation in an elderly or severely cognitively impaired subject at subset or when daylight is reduced”	Confusion, disorientation, agitation, screaming, delusional thinking, moaning, wandering	Circadian rhythm disturbance, sleep cycle changes	Lighting and sensory evaluation, structured environment, visual/verbal cueing, psychiatric and neuropsychological consultations	Background, assessment, intervention
(Bliwise, 1994)	“The phenomenon of agitation seemingly caused by, or at least strongly associated with, darkness”	Agitation	Circadian rhythmicity dysfunction	N/A	Sundowning existence and mechanisms
(McGaffigan & Bliwise, 1997)	“Agitation in dementia patients that has specific temporal exacerbation during the early evening or nocturnal hours”	Vocalization, wandering, combativeness	N/A	Pharmacological (antipsychotics, antidepressants) and alternative treatments (melatonin, light therapy)	Pharmacological and alternative treatments
(Dewing, 2003)	“The collective effects of cognitive and behavioural changes in persons with dementia that take place, for a variety of reasons, around late afternoon to nightfall”	Confusion, agitation, delirium, restlessness, wandering, screaming	Sleep disturbances, circadian rhythm issues, delirium, over-stimulation	Pharmacological and environmental interventions, therapeutic approaches	Sundowning behaviors and management strategies
(Wick & Zanni, 2005)	“Accidents, wandering, falls, and aggressive outbursts that occur between 4:00 p.m. and 8:30 p.m.”	Confusion, agitation, aggression, loud/repetitive verbalization, combativeness	Delirium, sensory and sleep disturbances, environmental factors	Light therapy, stimulation during sunset, memory aids, medication (antidepressants, antipsychotics)	Differential diagnosis, causes, treatment
(Bachman & Rabins, 2006)	“Delirium and agitation” within 1 hr of darkness or “the appearance or exacerbation of behavioral disturbances associated with the afternoon and/or evening hours”	Behavioral and neuropsychiatric symptoms	Nighttime unmet needs, disturbed sleep, circadian rhythm disruption	Environmental strategies (light therapy, activity structuring), pharmacological options (hypnotics, antipsychotics)	Definitions, prevalence, causes, treatments
(Sharer, 2008)	“Any disruptive behaviors in the late afternoon or early evening hours”	Increased anxiety, confusion, delusions, restlessness, wandering	Circadian rhythm abnormalities, melatonin decline	Strict schedules, exercise programs, pharmacological treatments (melatonin, benzodiazepines)	Sundowning theories, management strategies
(Khachiyants et al., 2011)	“A common clinical phenomenon manifested by the emergence or increment of neuropsychiatric symptoms in the late afternoon, evening or at night”	Confusion, disorientation, anxiety, agitation, aggression, pacing, wandering	Sensory deprivation, circadian and sleep disorders, environmental stressors	“S-M-A-R-T” and “P.I.E.c.e.S.” strategies, light therapy, pharmacological treatments (AChEIs, antipsychotics)	Epidemiology, etiology, diagnosis, treatment, prognosis, prevention
(Yevchak et al., 2012)	“A constellation of behavioral (motor and verbal) symptoms with onset or exacerbation in the later afternoon or evening hours, especially in individuals with pre-existing cognitive impairment, such as dementia”	Behavioral symptoms: wandering, restlessness, agitation, vocalizations	Cognitive impairment, routine changes, caregiver stress	Non-pharmacological (music therapy, light interventions, caregiver training), pharmacological treatments (melatonin, donepezil)	Definition, prevalence, antecedent factors, consequences, caregiver recommendations
(Cipriani et al., 2015)	“A wide range of neuropsychiatric symptoms often occurring in individuals with dementia”	Increased disorientation, confusion, agitation, restlessness, wandering, anxiety	Neurobiological and physiological factors, environmental and psychosocial factors	Non-pharmacological (light, noise management, multisensory stimulation), pharmacological treatments (cholinesterase inhibitors, antipsychotics)	Nosology, clinical characteristics, management
(Canevelli et al., 2016)	“Emergence or worsening of neuropsychiatric symptoms (NPS) in the late afternoon or early evening”	Neuropsychiatric symptoms	Neurobiological, pharmacological, physiological factors, environmental stressors	Non-pharmacological (light therapy, stable routines), pharmacological interventions (melatonin, cholinesterase inhibitors)	Definition, pathophysiology, clinical approach
(Gnanasekaran, 2016)	“The phenomenon of disruptive behavior that worsens in late afternoon in elderly institutionalized patients”	N/A	Circadian rhythm changes, hormonal and environmental factors	Therapeutic approaches including melatonin, cholinesterase inhibitors, environmental adjustments	Understanding and managing disruptive behavior
(Boronat et al., 2019)	“A cyclic delirium-like condition affecting the older population around the sunset hour that may last for a few hours”	Psychomotor, cognitive, speech disturbances; psychosis; affective and nonspecific symptoms; sleep changes	Circadian rhythm alterations, neurobiological and environmental factors	Pharmacological (antidepressants, antipsychotics, hypnotics) and non-pharmacological interventions (light therapy, occupational therapy)	Definitions, risk factors, pathophysiology, management
Xu et al., 2025 (Ours)	The onset or worsening of symptoms typically occurs in the late afternoon or evening, often aligned with local sunset times. These symptoms are assessed through various sensors and structured questionnaire-based observations. This condition is characterized by specific criteria, which may include (1) greater symptom severity in the evening compared to the morning, (2) symptoms that appear or intensify exclusively in the evening, or (3) peak symptom intensity during the evening. SS primarily affects individuals with dementia and Alzheimer’s disease but may also affect other older adults or those in specialized care settings	Psychomotor symptoms (e.g., agitation, aggression, combativeness, pacing, wandering), cognitive and perceptual disturbances (e.g., confusion, disorientation, delirium), mood and affective symptoms (e.g., depression, anxiety, euphoria, irritability, mood lability), psychosis (e.g., delusions, hallucinations, illusions), changes in activities of daily living (ADLs) and changes in instrumental activities of daily living (IADLs) (e.g., appetite changes, sleep disturbance, resistance behavioral, hoarding)	N/A	Using multimodal sensors to aid in risk identification, diagnosis, monitoring, and treatment	Characteristics of included studies, SS measurements in previous studies (summarizing both questionnaire-based methods and sensor-based methods), Multimodal data recording platform for SS

*Note:* N/A =  Not available or not applicable.

**Box 1:** | BEST-guided multimodal solutions for measuring SS. Following the Biomarkers, Endpoints, and other Tools (BEST) glossary introduced by the FDA-NIH Biomarker Working Group (Cagney et al., 2017; FDA-NIH Biomarker Working Group, 2016), which features biomarkers measured along the clinical continuum from pre-diagnosis and pre-treatment to post-treatment phases, we borrow this concept to help organize and identify several of the most recognized stages where multimodal solutions could be utilized in SS. In the following table, we first list the original definition of relevant biomarkers defined by the BEST glossary and then on the right column, the specific signs or situations that we are interested in using in our framework through multimodal solutions.

Category	Original Definition	Our Application
Risk identification	A biomarker that indicates the potential for developing a disease or medical condition in an individual who does not currently have clinically apparent disease or the medical condition	A multimodal solution can identify potential risk factors for developing SS in individuals who do not yet exhibit obvious symptoms, aiding in early intervention and environmental adjustments
Diagnosis	A biomarker used to detect or confirm presence of a disease or condition of interest or to identify individuals with a subtype of the disease	Multimodal solutions can detect or confirm SS by integrating data from various sources, providing a more accurate and comprehensive diagnosis than traditional observational methods
Monitoring	A biomarker measured serially for assessing status of a disease or medical condition or for evidence of exposure to (or effect of) a medical product or an environmental agent	Continuous monitoring of patients with SS using multimodal solutions can track disease progression, patient status, and response to environmental changes, allowing for timely interventions
Treatment/response	A biomarker used to show that a biological response has occurred in an individual who has been exposed to a medical product or an environmental agent	Multimodal solutions can evaluate the effectiveness of treatments for SS by monitoring both behavioral and biological responses, providing a comprehensive view of patient progress and treatment outcomes. In addition, multimodal solutions could serve as a treatment in themselves by combining two or more pharmacological and/or non-pharmacological interventions simultaneously for better efficacy

## REVIEW METHOD

A comprehensive literature search was conducted across multiple databases, including Web of Science, PubMed, Medline, APA PsycInfo, and IEEE Xplore, to identify studies relevant to SS. The search strategy employed the terms (“sundowning” OR “sundowning syndrome” OR “sundown syndrome”) across all fields. The initial inclusion criteria required that papers be either review articles or empirical studies addressing any aspect of SS, such as observations, interviews, interventions, or treatments. We restricted our search to papers published before July 2024, written in English, and with full texts available. This search yielded a total of 369 papers: 129 from Web of Science, 117 from PubMed, 52 from Medline, 3 from IEEE Xplore, and 82 from APA PsycInfo. Using Zotero Reference Manager, duplicates were removed, resulting in 196 unique papers. These were further screened based on abstracts, titles, and the availability of the full text, resulting in the exclusion of 49 articles due to irrelevance to SS or inaccessible full texts, leaving a total of 147 papers.

**Fig. 1:**
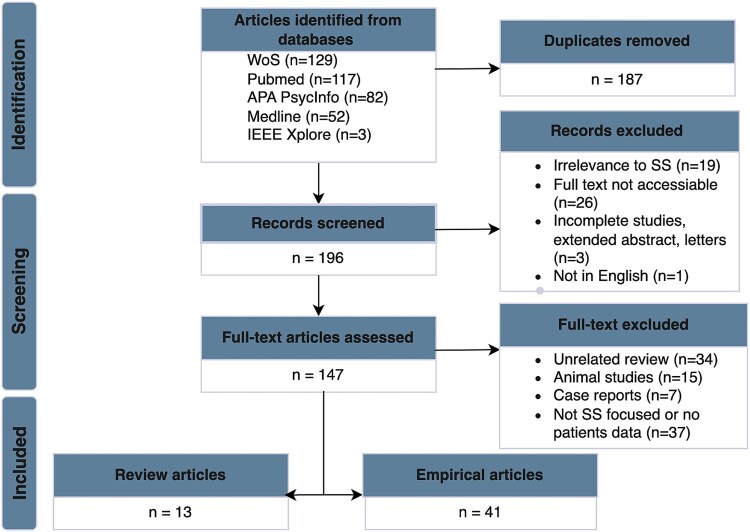
Flow diagram of the literature search.

In the second round, review and empirical studies were assessed separately. For review articles, inclusion was based on a focus on SS, excluding those primarily centered on other domains but mentioning SS briefly (e.g., reviews on melatonin or differential diagnoses such as delirium, as cited in Heimberger & Lukas, 2023). Empirical studies were included if they involved human participants, measured characteristics of SS, observed patients during sundowning times as a specific criterion, or sought treatments for SS. Some studies on delirium and agitation were retained due to the challenge of distinguishing these symptoms from SS, especially when studies explicitly chose sundowning times as observation or measurement windows for these symptoms. In this regard, we further excluded 34 unrelated review papers, 15 animal studies, 7 case reports, and 37 papers irrelevant to the study’s conceptual framework or lacking specific assessment and measurement methods. After the second round, 13 review papers and 41 empirical papers remained. Our review protocol, developed in accordance with the established guidelines for conducting scope literature reviews as outlined in Tricco et al. (2018), is illustrated in Fig. 1. The specific reasons for the exclusion of each paper are provided in Supplementary Material Table 1. This study was not pre-registered. However, in adherence to the requirements of transparency and openness, all supplementary materials are available at the study’s project with the following link: https://osf.io/y9ew8/?view_only=f8e8a77261e749eab44d41d8e6db6c77.

## RESULTS

### Characteristics of included review studies

Table 1 provides a summary of the review papers included in the current analysis. Papers not included were primarily excluded due to irrelevant content and/or lack of systematic data summarization. Among the remaining review papers, three studies published between 1991 and 2000 (23.08%), four between 2001 and 2010 (30.77%), and six from 2011 to 2020 accounted for 46.15%. Many studies broadly defined SS as a range of disruptive behaviors or neuropsychiatric symptoms (NPS) without specifying particular symptoms (Canevelli et al., 2016; Cipriani et al., 2015; Dewing, 2003; Gnanasekaran, 2016; Khachiyants et al., 2011; Sharer, 2008; Yevchak et al., 2012), whereas others focused on specific symptoms such as agitation, delirium, and wandering (Bachman & Rabins, 2006; Bliwise, 1994; Boronat et al., 2019; Duckett, 1993; McGaffigan & Bliwise, 1997; Wick & Zanni, 2005). Four studies specifically defined SS in patients with dementia (PWD), whereas others considered a general older population or concentrated solely on manifested symptoms without a targeted population (Cipriani et al., 2015; Dewing, 2003; McGaffigan & Bliwise, 1997; Yevchak et al., 2012). Additionally, three studies did not specify the time of day for symptom manifestation (Bliwise, 1994; Cipriani et al., 2015; Duckett, 1993), five defined it around sunset (Boronat et al., 2019; Canevelli et al., 2016; Gnanasekaran, 2016; Sharer, 2008; Yevchak et al., 2012), and three included night hours (Dewing, 2003; Khachiyants et al., 2011; McGaffigan & Bliwise, 1997). Two studies pinpointed specific times for SS, such as 4:00 p.m. to 8:30 p.m. (Wick & Zanni, 2005) or within 1 hr of darkness (Bachman & Rabins, 2006). The prevalence rates reported varied from 2.4% to 66%, with some studies reporting the prevalence based on different patient groups (Bachman & Rabins, 2006; Boronat et al., 2019; Sharer, 2008; Wick & Zanni, 2005).

By observing the key structures listed in column 6 (“Main Content”) of Table 1, we have summarized the main content related to “Symptoms & Characteristics,” “Pathological Analysis,” and “Recommended Solutions” from previous studies. As these topics are not the primary focus of the current review, we highlighted only a few key reviews in each category for future research reference. Boronat et al. (2019), for instance, identified a total of 206 symptoms from previous studies, categorizing them into seven clusters: “psychomotor alterations,” “cognitive disturbance,” “speech disturbances,” “psychosis,” “affective symptoms,” “nonspecific symptoms,” and “changes in sleep patterns” (Boronat et al., 2019). The most frequently reported symptoms were psychomotor issues such as agitation, aggression, and restlessness, along with cognitive disturbances like confusion and disorientation. For pathological analysis, many studies highlighted alterations in circadian rhythms, with recent reviews providing detailed analyses of combined influences from biological, psychological, and environmental factors (Canevelli et al., 2016; Cipriani et al., 2015; Gnanasekaran, 2016; Khachiyants et al., 2011; Wick & Zanni, 2005). For treatment, both pharmacological and non-pharmacological methods have been explored and summarized in previous studies (Canevelli et al., 2016; Cipriani et al., 2015; Dewing, 2003; Duckett, 1993; Gnanasekaran, 2016; Khachiyants et al., 2011; McGaffigan & Bliwise, 1997; Sharer, 2008; Yevchak et al., 2012). For details, please refer to Table 1 and the relevant papers. For a detailed list of abbreviations used in this paper, please refer to Appendix A.

### Characteristics of included empirical studies

**Fig. 2:**
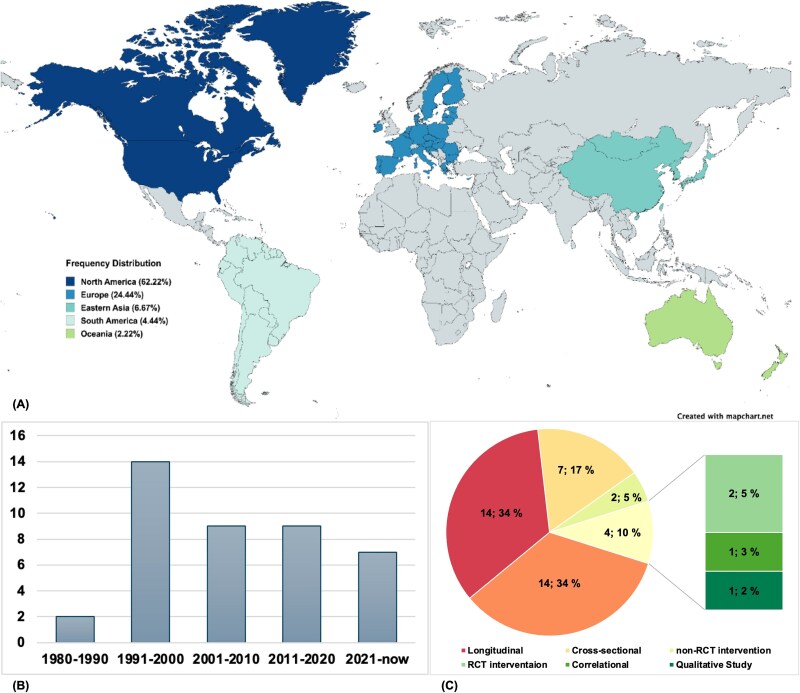
Main characteristics and demographic information of the included empirical studies. (A) Distribution of publications based on continent/area; (B) number of publications by decade; (C) pie chart showing the proportional distribution of different types of studies, with each label indicating the count followed by the percentage of the total (e.g., 14; 34% indicates 14 studies, accounting for 34% of the total). The map in (A) was created using MapChart (mapchart.net) and is licensed under a Creative Commons Attribution-ShareAlike 4.0 International License.

Figure 2 illustrates the main characteristics and demographic information of the included empirical studies. In Supplementary Material Table 2, we present a comprehensive summary of 41 empirical studies investigating SS across various populations and settings. The table includes key study details including “Study,” “Year,” “Country & Region,” “Study design,” “Operational definition of SS,” “Defined occurrence time/Recorded time,” “Setting,” “Study population,” “Inclusion criteria,” “Exclusion criteria,” “Race/Ethnicity,” “Sample size,” “Sex (% Female),” “Age (mean, SD/range),” “Observation/Reported group,” “Behavioral assessment tool,” “Behavioral symptoms,” and “Other sensors (i.e., single- or multi-modality used).” Readers can refer to the table for more details. In brief, all studies were published between 1987 and 2023, with 2 studies (4.88%) published before 1990 and 14 studies (34.15%) published between 1991 and 2000. Additionally, there were nine studies (21.95%) published in each of the decades from 2001 to 2010 and from 2011 to 2020. A new trend has recently emerged, with seven studies (17.07%) published from 2021 up to 2023. Most studies, totaling 39, were conducted in the Northern Hemisphere, with only three studies undertaken in the Southern Hemisphere. The predominant countries of publication are the USA, Canada, and European countries such as France and Italy, with only three studies from Asia, two from South America, one from Australia, and none from Africa. Within the USA, studies are mainly distributed across the East Coast, West Coast, and Midwest, with the majority conducted in the Maryland and Washington, D.C. area (6 studies, 25%) and California (5 studies, 20.83%). The most common study types were longitudinal (14 studies, 34.15%) and cross-sectional studies (14 studies, 34.15%), relying on nurses’ or caregivers’ observations and reports. More non-randomized controlled trial (RCT)interventions (7 studies, 17.07%) were conducted compared to better-controlled RCT interventions (2 studies, 4.88%), with many using convenient methods to recruit patients and involving only a few patients in the targeted sample. The rest of the studies employed designs such as correlational, qualitative, and cohort studies, collectively contributing to ~10% of the total research.

As for the population, sample sizes vary significantly, with some studies including <10 samples, whereas others have >100, reaching up to 497 samples. The focus has been on AD patients (22 studies, 53.66%), 13 studies on patients with dementia in general without targeting a specific type of dementia, and a few studies on cognitive impairment patients, including mild cognitive impairment (3 studies, 7.32%) and other types of patients requiring special care (3 studies, 7.32%). The majority of studies did not report participants’ racial or ethnic backgrounds (31 studies, 68.29%). In nine studies (21.95%), a significant portion involved predominantly participants of European descent, whereas only one study (2.44%) primarily focused on participants who self-identified as being of African descent. More studies recruited female participants (28 studies, 65.85%) than male participants. The included populations involve older adults from ~50 up to ~100 years old, with most patients aged 70–90 years.

### Definition of sundown syndrome

Theoretically, Evans (1987) defined SS as the “appearance or exacerbation of symptoms of confusion associated with the late afternoon or early evening hours” (Evans, 1987). Subsequent studies have generally used a similar definition but have modified or added symptoms, or specified it for dementia or AD patients (for a review, see Yevchak et al., 2012). However, due to the complexity of SS and the limitations of research, it is still difficult to provide an accurate definition. Therefore, we believe that before concluding the theoretical definition, it is more important to clearly operationally define SS. Moreover, we argue that many discrepancies in previous studies, such as the presence or absence of SS and the wide range of prevalence rates, are due to unclear operational definitions. Thus, we have summarized the operational definitions from previous articles and categorized them into the following types: (1) symptoms are larger or more pronounced in the evening compared to the morning; (2) symptoms are only present in the evening; (3) symptoms peak in the evening (see Supplementary Material Table 2 for more details). There was also a wide variation in the time settings, with some studies only including the evening hours, approximately from 15:00 to bedtime, some encompassing a broader range of afternoon hours, and others extending into the whole night. Although most studies collected data from patients with dementia, some were limited to dementia or even just AD populations, whereas others did not specify and included a broader group of elderly people or those requiring special care. Building on this understanding, we propose a refined operational definition (see Table 1) for SS that includes specific evening time frames based on local sunset times, clearly listed symptoms that appear or intensify in the evening, and criteria for symptom frequency and severity. We also recommend adopting a systematic screening tool, such as a structured questionnaire specifically designed to assess SS, which we will introduce in the next section.

### Sundown syndrome measurements in previous studies

#### Questionnaire-based subjective measurements

Questionnaires are the most direct method for measuring SS, but the choice of questionnaire is crucial. Early research was significantly influenced by researchers’ definitions and interests regarding SS, often leading to measurements focused on one or a few specific symptoms. For example, many early studies regarded SS as evening delirium or agitation and used questionnaires such as the Confusion Inventory (Evans, 1987) or the Agitation Behavior Mapping Instrument (ABMI; Cohen-Mansfield et al., 1989) to measure or screen SS patients. However, as highlighted before, SS consists of various symptoms. These single-symptom targeted questionnaires may overlook some behaviors or symptoms during observation or interviews, leading to the misdiagnosis or underdiagnosis of some SS patients during the screening process.

In this context, the Neuropsychiatric Inventory (NPI; Cummings et al., 1994) has been used and validated in several later studies (Dechamps et al., 2008; Menegardo et al., 2019; Sevilla et al., 2018; Trumpf et al., 2023; Venturelli et al., 2013, 2016). The NPI is a structured interview designed to assess a broad spectrum of 12 neuropsychiatric symptoms, including delusions, hallucinations, agitation/aggression, depression, anxiety, euphoria, apathy, disinhibition, irritability, aberrant motor behavior, nighttime behavior disturbances, and appetite changes. Although the NPI offers a relatively comprehensive assessment compared to single-symptom targeted questionnaires, it falls short in specifically targeting the unique characteristics of SS, particularly the timing of symptom onset.

To address this gap, more specific tools capturing the temporal dynamics of SS have been developed, such as the Time-Based Behavioral Disturbance Questionnaire (Bliwise et al., 1992) and the Sundowning Behavior Flowsheet (Wallace, 1994). These tools aim to capture the temporal dynamics of disruptive behaviors relating to SS. Additionally, the Full Listening Log used in Lineweaver et al. (2022a, 2022b), specifically designed to measure the severity of several key SS symptoms improvement during music therapy, also coded a variety of behavioral changes, including alterations in demeanor, eye movements, body language, vocal sounds, and overall movement during therapy, which could be used in combination with the multimodal solutions we proposed. Furthermore, several more structured questionnaires have been developed. The Sundown Syndrome Questionnaire (SSQ; Pyun et al., 2019), for instance, is a caregiver-completed tool that assesses SS behavior. It consists of four parts and takes ~15 min to complete. Part A assesses 11 behavioral problems, with scores ranging from 0 to 11. Part B determines if these behaviors worsen in the afternoon or evening, whereas Part C identifies specific time periods of symptom aggravation. Part D evaluates the frequency of the most prevalent behavioral problem. The final severity score, Total B, is calculated by multiplying the total score from Part A by the frequency score from Part D, with a range of 0–44. The Sundowning Evaluation Questionnaire (SEQ; Toccaceli Blasi et al., 2023), inspired by the NPI, is also administered to caregivers and focuses on neuropsychiatric symptoms that occurred in the previous month. It begins with a screening question to detect SS, followed by 12 additional questions to further characterize the behavioral disturbances associated with the syndrome. Although there is some overlap in the symptoms assessed by both questionnaires, such as anxiety, depression, delusions, and hallucinations, the SEQ is more focused on mood-related behaviors such as irritability, anxiety, depression, apathy, and euphoria, whereas the SSQ covers a wider spectrum of symptoms, including cognitive disorientation and motion-related symptoms.

To provide readers with a more direct comparison of the various questionnaires used to assess SS, an overview of various assessment tools is listed in Table 2. Given the broad range of symptoms associated with SS, this table presents a brief description of the different instruments used in research and highlights the specific aspects of the condition they targeted (see Columns 5 and 6). Future studies could benefit from combining the strengths of these tools to develop a more comprehensive instrument that covers the full spectrum of SS symptoms.

**Table 2:** The overview of assessment tools used in sundown syndrome measurement

Abbreviation	Name	Questionnaire Developed by	Used in Studies	Focused Symptoms/Syndrome	Symptom Category	Description
CI	Confusion Inventory	(Evans, 1987)	(Evans, 1987)	Confusion	CPD	A checklist assessing 48 indicators of confusion. It compares morning and afternoon scores to identify symptoms, with higher scores indicating increased confusion
NEECHAM	NEECHAM Confusion Tool	(Neelon et al., 1996)	(Jagmin, 1998)	Confusion	CPD	Evaluates confusion through observation of processing ability, behavior, and physiological conditions in clinical settings. Scores are derived from direct assessment, with higher scores indicating better function
CAM	Confusion Assessment Method	(Inouye et al., 1990)	(Silva et al., 2017)	Confusion	CPD	Identifies delirium in hospitalized patients through structured assessment of four features and uses a diagnostic algorithm to determine delirium presence
ABRS	Agitated Behavior Rating Scale	(Bliwise et al., 1993)	(Bliwise et al., 1993; Lebert et al., 1996; Martin et al., 2000)	Agitation	PS	Measures agitation across five categories: manual manipulation, searching/wandering, escape behaviors, tapping/banging, and verbal agitation. Intensities are scored, with higher scores indicating greater agitation severity
ABMI	Agitation Behavior Mapping Instrument	(Cohen-Mansfield et al., 1989)	(Cohen-Mansfield, 2007; Cohen-Mansfield et al., 1989, 1992)	Agitation	PS	Maps the presence and intensity of 15 specific agitated behaviors on an hourly basis during an 8-hr shift. Scores reflect agitation severity across different shifts
CMAI	Cohen-Mansfield Agitation Inventory	(Cohen-Mansfield, 1986)	(Richards et al., 2021; Shih et al., 2017, 2020)	Agitation	PS	Assesses agitation in dementia patients, consisting of 29 items that rate the frequency of behaviors on a 7-point Likert scale. Scores are summed to indicate overall agitation severity, with higher scores indicating greater agitation
NPI	Neuropsychiatric Inventory	(Cummings et al., 1994)	(Dechamps et al., 2008; Menegardo et al., 2019; Sevilla et al., 2018; Trumpf et al., 2023; Venturelli et al., 2013, 2016)	Delusions, hallucinations, agitation/Aggression, depression, anxiety, euphoria, apathy, disinhibition, irritability/lability, aberrant motor behavior, nighttime behavior disturbances, appetite and eating abnormalities	Multiple (All)	Evaluates 12 neuropsychiatric symptoms in routine clinical practice settings. Scores are based on caregiver interviews and observations, reflecting symptom severity and impact on daily living
TBDQ	Time-Based Behavioral Disturbance Questionnaire	(Bliwise et al., 1992)	(Bliwise et al., 1992; Gallagher-Thompson et al., 1992)	Combativeness, agitation and purposeless movement, wandering, incoherent speech, hallucination, confusion, disorientation	Multiple (PS, CPD, PSY)	Assesses temporal patterns of the seven most typically disruptive behaviors in cognitive disorders through caregiver reports. The questionnaire is scored to evaluate frequency and severity over time, aiding in understanding behavior patterns
FLL	Full Listening Log	(Lineweaver et al., 2022a)	(Lineweaver et al., 2022a; Lineweaver et al., 2022b)	Confusion, restlessness, agitation, aggression, disengagement, repetitiveness, unresponsiveness	Multiple (CPD, PS, MAS)	Evaluates the impact of music listening on seven SS before and after each music session. Records session details and behavior changes, with higher scores indicating improved functioning
SBF	Sundowning Behavior Flowsheet	(Wallace, 1994)	(Wallace, 1994)	Verbal outbursts, wandering, acts of violence toward self or others, upset behavior or agitation, resistance or refusal of core	Multiple (PS, MAS)	Uses a subset of “The Nursing Home Behavior Problem Scale” to document the presence and frequency of five specific behavioral changes in nursing home residents
SSQ	Sundown Syndrome Questionnaire	(Pyun et al., 2019)	(Pyun et al., 2019)	Disorientation to time, place, or person, anxiety, depression, wandering, aggression, emotional lability, hoarding behavior, delusion, hallucination	Multiple (All)	A caregiver-completed tool for assessing SS in cognitive disorders, consisting of four parts. Scores range from 0 to 44, evaluating behavior problems, symptom worsening in the evening, and frequency, providing a comprehensive severity score for symptom management
SEQ	Sundowning Evaluation Questionnaire	(Toccaceli Blasi et al., 2023)	(Toccaceli Blasi et al., 2023)	Delusions, hallucinations, agitation/Aggression, depression, anxiety, euphoria, apathy, disinhibition, irritability/lability, aberrant motor behavior, nighttime behavior disturbances, appetite and eating abnormalities.	Multiple (All)	An NPI-based questionnaire to assess neuropsychiatric symptoms during afternoon-evening hours. Includes an initial screening question followed by 12 questions to detail neuropsychiatric symptoms observed in the past month. Participants are classified as sundowners or non-sundowners based on their responses

*Notes:* The symptom categories in Column 5 refer to the classifications discussed in Chapter 4: “PS” (psychomotor symptoms), “CPD” (cognitive and perceptual disturbances), “MAS” (mood and affective symptoms), “PSY” (psychosis), and “ADL/IADL” (ADL/IADL changes). “Multiple” indicates a combination of these categories for instruments that assess multiple symptoms

#### Sensor-based objective measurements

As we just introduced, SS is primarily assessed through observational ratings or reports from nurses and caregivers. Although these subjective assessments provide valuable insights, they are often limited by biases and inconsistencies. To address these limitations, researchers have explored objective measurements using various behavioral and biological sensors, which may offer more reliable indicators of SS. In this section, we review the evolution of measurement methods for SS, from single-modal to emerging multimodal approaches.

##### Single-modal measurements

One of the most used objective measures of SS is activity monitoring, often used alongside questionnaire-based methods. Rindlisbacher and Hopkins (1992), for instance, were pioneers in this area, employing ambulatory monitors with motion sensors to continuously track activity in 12 AD patients over 4 days. They observed heightened activity in the afternoon, especially in patients with mid-stage dementia. However, discrepancies were noted between movement data and nursing classifications of “sundowners” and “non-sundowners,” suggesting that activity measurements alone may not fully capture SS symptoms (Rindlisbacher & Hopkins, 1992). Subsequent studies, such as those by Ghali et al. (1995), extended this methodology by analyzing 48-hr activity data and classifying patients based on disease progression. Their findings indicated that peak activity times mainly varied with the progression of the disease, with patients becoming most active later in the day as the disease advanced (Ghali et al., 1995). Similarly, Mahlberg et al. (2004) used actimetry to substantiate clinical observations and assess the effects of melatonin treatment for SS. Their findings showed that five out of seven patients experienced a significant reduction in nocturnal activity between 9 p.m. and 6 a.m., highlighting the potential of actimetry as an objective tool to monitor treatment effects in SS as well (Mahlberg et al., 2004). More recent work by Trumpf et al. (2023) utilized a sophisticated hybrid motion sensor worn on the lower back, which allowed for a nuanced analysis of postural events and activity levels in 73 patients over 48 hr. They identified 34% of patients as sundowners based on their active periods, aligning with nursing staff observations (Trumpf et al., 2023).

Physiological measurements provide an additional layer of insight by examining bodily functions that may correlate with the variation of SS. For instance, Venturelli et al. (2013) found significantly higher cortisol levels in AD patients exhibiting SS symptoms at sunset compared to other times of the day, suggesting a potential link between glucocorticoid dysregulation and SS (Venturelli et al., 2013). A subsequent study by the same group (Venturelli et al., 2016) demonstrated that interventions such as aerobic exercise, monitored using activity and heart rate sensors, could reduce SS symptoms through the downregulation of cortisol levels. However, it is unfortunate that the collected activity and heart rate data were not utilized to directly assess SS, limiting the potential insights that could have been gained from these measurements. Recent advancements have also extended the measurement of SS to the molecular level. For instance, Pyun et al. (2019) reported that AD patients with SS were more likely to carry the Apolipoprotein E4 (APOEɛ4) allele, which was associated with more severe disease progression, rapid eye movement sleep behavior disorder, and higher dementia ratings (Pyun et al., 2019). Neurophysiological and neuroimaging techniques, though less commonly employed, provide significant insights into brain activity associated with SS. Rabi et al. (2022) utilized electroencephalography (EEG) to examine daily fluctuations in inhibitory control among individuals with amnestic mild cognitive impairment. Their results revealed heightened inhibitory deficits in the evening, suggesting that neuroimaging methods may serve as valuable biomarkers for identifying the neural mechanisms underlying SS (Rabi et al., 2022).

Despite the utility of single-modal approaches, each method has distinct limitations. For instance, although activity measurements are valuable for monitoring physical changes, they do not adequately capture cognitive and emotional fluctuations associated with SS. Moreover, distinguishing between normal activity variations and SS-specific patterns remains difficult without additional contextual data. Early studies (e.g., Ghali et al., 1995; Rindlisbacher & Hopkins, 1992) often relied on labor-intensive manual filtering processes to exclude unrelated activities, which could compromise data accuracy. Similarly, physiological measurements, such as hormonal analyses, may not directly link to specific behavioral manifestations of SS, thereby limiting their diagnostic utility.

##### Emerging multimodal approaches

To overcome the limitations of single-modal methods, recent studies have explored multimodal approaches that integrate data from various sources. For instance, although both sensors focused on activity-related data, Paillard et al. (2016) used a force platform and gait analysis device to examine balance and gait changes in AD patients during the evening. Their findings revealed that balance control and walking were significantly altered during evening hours, highlighting the potential of using these multimodal data to improve the detection of fall risks among AD patients (Paillard et al., 2016). Additionally, Volicer et al. (2001) monitored dynamic changes in core body temperature and motor activity over 72 hr in AD patients with and without SS, as well as healthy controls. They found that the severity of SS was associated with a delayed temperature peak, a diminished correlation between circadian temperature rhythms and the 24-hr cycle, and a reduced amplitude of the temperature curve (Volicer et al., 2001). These findings suggest that integrating core body temperature data with activity monitoring could better reflect the disrupted circadian rhythms inherent in SS.

Richards et al. (2021) further underscored the value of multimodal approaches by examining nighttime agitation behaviors, sleep minutes from actigraphy, and fasting morning blood samples for iron status in a clinical trial of PWD. Their multivariable model showed that sleep minutes and transferrin saturation percentage were negatively associated with the frequency of nighttime agitation behaviors, explaining 20% of the variance. This suggests that treating iron deficiency could help decrease nighttime agitation and improve sleep and overall quality of life in PWD (Richards et al., 2021).

Furthermore, Wilks et al. (2021) utilized cerebrospinal fluid (CSF) biomarkers, specifically beta-amyloid (Aβ42) and phosphorylated tau181 (pTau), to differentiate between CSF “Negative” and CSF “Positive” participants. They combined these biological measures with smartphone-based cognitive assessments, tracking participants’ associative memory, processing speed, and visual working memory four times per day over seven consecutive days at quasi-random time intervals. This innovative combination of biological and behavioral data provided a comprehensive view of how AD pathology affects cognitive performance throughout the day. Notably, older adults with CSF-confirmed AD pathology exhibited subtle impairments in associative memory during evening hours, but not in the morning, providing a preliminary biomarker of cognitive symptoms for SS (Wilks et al., 2021).

Environmental factors such as light exposure and noise levels are also known to significantly influence SS (Bliwise et al., 1993; Toccaceli Blasi et al., 2023), yet direct measurements of these variables are rarely included in SS studies. In the study by Martin et al. (2000), activity and light exposure data were continuously collected to measure both agitation rhythms and light exposure patterns. The results indicated that the mean acrophase for agitation was 14:38, with substantial variability in timing (*SD* = 3 hr 23 min, range = 01:26–23:44). However, only 2 patients (2.4%) showed the most severe agitation during evening hours. Although the limited sample size made it challenging to fully characterize SS, the inclusion of environmental measurements revealed that overall light exposure in the group was extremely low. Higher levels of nighttime illumination were associated with less pronounced agitation rhythms and lower amplitude rhythms (Martin et al., 2000), highlighting the potential of multimodal approaches, including environmental monitoring and light therapy, to help mitigate agitation symptoms in SS.

In summary, the methods for measuring SS have evolved significantly. Early approaches primarily focused on assessing single symptoms, but as the understanding of SS has grown, the focus has shifted toward capturing multiple symptoms simultaneously. Alongside this shift, sensor technology has progressed from single-modality systems, which capture only one type of data for certain symptoms, to more advanced multimodal systems that integrate, for example, activity, physiological, and environmental data. However, despite these advancements, current studies are still limited by small sample sizes and integration of limited data streams, preventing a full understanding of SS’ multifaceted nature. To overcome these limitations, the next chapter presents a more comprehensive multimodal framework for measuring SS. This framework not only integrates different types of sensors but also links them directly to the specific symptoms and applications relevant to SS, offering a more complete and practical approach to managing this complex condition.

## MULTIMODAL DATA RECORDING PLATFORM FOR SUNDOWN SYNDROME

Measuring SS is inherently complex due to the diverse and multifaceted nature of its symptoms, coupled with the limited understanding we currently have. No single modality can adequately capture all aspects of SS, as its various symptoms manifest in unique ways, requiring tailored measurement approaches and tools. Based on our observations from Table 2 and building on the symptom categories proposed in previous work, such as the work of Boronat et al. (2019), who highlighted “psychomotor,” “cognitive,” “speech disturbance,” “psychosis,” “affective symptoms,” “nonspecific symptoms,” and “changes in sleep” (Boronat et al., 2019), as well as the classification by Van Der Linde et al. (2014), which includes “affective symptoms,” “psychosis,” “hyperactivity,” and “euphoria” (Van Der Linde et al., 2014), we have refined the symptom categorization of SS into five primary categories. These categories, which can be examined using comparable sensor technologies, include (1) psychomotor symptoms, (2) mood and affective symptoms, (3) psychosis, (4) cognitive and perceptual disturbances, and (5) changes in ADL and IADL.

Although categorizing SS symptoms into these five primary domains provides a foundational structure for understanding the condition, a further challenge lies in the practical application of this framework in real-world contexts. It is not enough to simply identify and classify symptoms. Measurement strategies must also be adapted to address specific practical applications and downstream tasks associated with each symptom category. By understanding the relationships between these symptoms and their corresponding real-world factors, we can offer more targeted support for both patients and caregivers. In the following sections, as outlined in Box 1, we explore the application of various sensor technologies to effectively measure different symptom categories. For each category, we identify key actionable items and features that aid in recognizing risk factors, enabling diagnosis, supporting real-time symptom monitoring, and providing multimodal solutions for symptom management.

### Psychomotor symptoms

Psychomotor symptoms, characterized by abnormal and excessive motor activity such as agitation, aggression, combativeness, pacing, and wandering, have been identified as the most prevalent symptom cluster in individuals with dementia (Boronat et al., 2019). These behaviors often reflect heightened energy levels and a lack of motor control, making them particularly challenging to manage. Among them, agitation and pacing are frequently cited as some of the most distressing and dangerous symptoms, posing significant risks to both PWD and their caregivers. To better understand and track these symptoms, researchers have employed tools such as agitation assessment scales and actigraphy-based monitoring. Given their prevalence and impact, psychomotor symptoms represent a critical target for analysis within our multimodal solution platform.

For risk identification, behavioral data collected through actigraphy watches and ambient sensors, such as passive infrared (PIR) sensors and other environmental sensors that can monitor ambient light, sound, humidity, atmospheric pressure, and temperature, are invaluable. In a conceptual study by Au-Yeung et al. (2020), higher activity levels, fewer room transitions, lower humidity, and reduced light levels in bathrooms were identified as potential environmental triggers for agitation, distinguishing evenings before agitated nights from those without agitation (Au-Yeung et al., 2020). These findings suggest that activity levels, when combined with environmental factors, can significantly contribute to identifying potential risk factors for psychomotor disturbances.

Diagnosis of psychomotor symptoms can be enhanced by utilizing multimodal solutions that integrate both physiological and behavioral assessments. For instance, Iaboni et al. (2022) tracked agitation events in 17 dementia patients using the Empatica E4, a wearable device equipped with sensors to measure motion and physiological signals such as blood volume pulse, electrodermal activity (EDA), and skin temperature. Through the development of personalized models, the study effectively classified agitation events (e.g., motor agitation, verbal aggression, physical aggression), achieving a median area under the receiver operating curve (AUC) generally exceeding 0.8 (Iaboni et al., 2022). When combined with validated questionnaires, these technologies allow healthcare providers to accurately diagnose or differentiate between various forms of agitation or psychomotor symptoms. Additionally, ML algorithms enhance diagnostic accuracy by analyzing complex datasets to identify patterns indicative of specific psychomotor disturbances.

Continuous monitoring of psychomotor symptoms can be enhanced by combining actigraphy with ambient sensors. Studies using actigraphy have demonstrated significant differences in motor activity between low and high agitation groups (Knuff et al., 2019; Nagels et al., 2006), with actigraphy metrics showing strong correlations with CMAI and NPI scores (Knuff et al., 2019). In addition to wearable sensors, ambient technologies such as PIR motion sensors, laser-based systems like Emerald, and door contact sensors have been utilized to track patients’ movements, locations, and walking speeds. PIR motion sensors have proven particularly effective in detecting episodes of agitation (Au-Yeung et al., 2020). For behaviors such as pacing and wandering, which involve not only increased activity but also aimless movement, GPS modules are crucial for tracking outdoor wandering and preventing patients from becoming lost. Several approaches have been developed to detect unsafe wandering and guide patients back to safety (Hammoud et al., 2018; Hassan & Khan, 2019). Additionally, video recordings from single or multiple cameras offer comprehensive behavioral insights. For example, Khan et al. (2022) developed a spatiotemporal convolutional autoencoder trained on normal behaviors to detect agitation as an anomaly. When tested on video data from a dementia unit, the model achieved an AUC of 0.754, illustrating its potential for use in long-term care settings (Khan et al., 2022).

In terms of treatment, timely reminders or the administration of medications for agitation or other psychomotor symptoms, along with real-time technological interventions such as digital therapeutic screens or virtual reality, could help mitigate symptoms. For instance, exposure to dynamic nature-based digital interventions (Nicholas et al., 2023) and family video messaging (Waszynski et al., 2018) has been shown to reduce agitation. By combining the monitoring methods discussed and applying non-pharmacological interventions, healthcare providers could better manage psychomotor symptoms in real time.

In summary, multimodal systems that integrate data from wearable sensors, capturing both activity and physiological signals, along with ambient monitoring technologies and video surveillance, can provide a comprehensive view of a patient’s psychomotor symptoms. Activity data and video systems deliver detailed behavioral and contextual insights, allowing for the detection of subtle changes that may indicate emerging psychomotor disturbances. Moreover, physiological signals and environmental data enhance diagnostic accuracy and risk identification. Combined with advanced real-time analytics, it can generate alerts that allow caregivers to respond promptly, administer appropriate treatments, and assess intervention effectiveness, thereby enabling personalized adjustments to care plans.

### Cognitive and perceptual disturbances

Cognitive and perceptual disturbances, such as confusion, disorientation, and delirium, are hallmark clinical features of dementia and represent another critical domain that can be exacerbated during sundowning episodes. These symptoms often manifest as increased mental confusion, temporal and spatial disorientation, and episodes of delirium, particularly in the late afternoon or evening. Numerous studies have reported that individuals may exhibit increased confusion (Jagmin, 1998; Johnson, 1997; Lineweaver et al., 2022a, 2022b), disorientation in terms of time or space (Bliwise et al., 1992; Gallagher-Thompson et al., 1992; Pyun et al., 2019), and reduced inhibitory functioning (Rabi et al., 2022) in SS samples. Although few studies have directly applied sensor-based solutions specifically to measure SS, cognitive and perceptual disturbances are core characteristics of dementia and cognitive impairment. Consequently, many studies have used various sensors to automatically assess these disturbances or detect the presence of dementia.

Risk identification could be enhanced through the integration of long-term app-based questionnaires or cognitive ability games/tasks combined with wearable sensors capable of measuring electrocardiogram (ECG) and EDA. These tools provide insights into physiological changes that may precede cognitive episodes. For instance, Rykov et al. (2024) demonstrated significant and strong associations between physiological measures, primarily heart rate variability (HRV), and executive function composites using wearable data collected over a 10-week clinical trial (Rykov et al., 2024). The mobile app and wearable sensors can collect longitudinal data. By integrating cognitive tasks with wearable sensors and applying ML to analyze patient patterns, it is possible to build detailed patient profiles and identify those at risk of cognitive decline and SS. Additionally, language and speech abilities, such as word-finding difficulties, reduced verbal fluency, and subtle acoustic features (e.g., prosody, slow speech rate, frequent hesitations), as well as eye activity indicators like gaze-fixation instability and prolonged saccades latency (Anderson & MacAskill, 2013; Ionescu et al., 2023), are critical components in cognitive assessments. These have been used as digital biomarkers for the early detection of dementia (Li et al., 2022). Collecting these data longitudinally and integrating it with electronic health records, which contain vast amounts of patient-specific information, have shown potential in predicting dementia (Graham et al., 2020; Nori et al., 2019).

For diagnosis, standardized cognitive tests like the Mini-Mental State Examination (MMSE) or Montreal Cognitive Assessment (MoCA) provide a baseline assessment of cognitive function. This can be supplemented with data from neuroimaging techniques like EEG and MRI, which offer insights into brain function and capture changes in neural activity patterns associated with cognitive disturbances. For instance, previous studies have shown an average classification accuracy of 89% in distinguishing AD from healthy groups and 82% in detecting MCI using language features, such as spontaneous speech data, based on 33 eligible studies (Alberdi et al., 2016). Changes in EEG signals, such as increased power in low frequencies, decreased power in high frequencies, reduced pattern complexity, and decreased synchrony between different brain regions, are effective in identifying dementia (Alberdi et al., 2016; Cassani et al., 2018).

Multimodal monitoring systems that integrate wearable sensors, including speech recognition devices and physiological recording tools (Fouad & Labib, 2023; Li et al., 2022; Martínez-Nicolás et al., 2021), can provide real-time and precise insights into a patient’s cognitive status and used to assist in monitoring the cognitive abilities of PWD. For instance, based on 33 eligible studies, previous research has reported an average classification accuracy of 89% for detecting AD and 82% for detecting MCI from healthy groups, using language features such as spontaneous speech data (Petti et al., 2020). Additionally, an accuracy of 96.55% was achieved in detecting AD using only three central lobe EEG electrodes (Fouad & Labib, 2023), highlighting the potential for profiling cognitive abilities in dementia patients within more real-life settings.

The treatment of cognitive and perceptual disturbances involves both pharmacological and non-pharmacological strategies, and could be also enhanced by multimodal solutions. Multimodal technologies can mainly facilitate these approaches by providing comprehensive insights into patient needs and treatment efficacy. For example, various digital applications and assistive technologies (Yousaf et al., 2020) have been developed to support cognitive training and enhance communication between patients and caregivers. Additionally, a review of multimodal non-pharmacological interventions showed that in 90% of the studies, participants were reported to experience cognitive improvement, stability in their dementia, or a delay in cognitive decline, which could complement the shortcomings of previous pharmacological treatments (Chalfont et al., 2020).

Overall, although most methods and features discussed here are typically used to detect dementia or cognitive impairment in general rather than specific cognitive symptoms in SS, we recognize that wearable physiological recordings, combined with behavioral indicators such as speech features and eye movements, could provide valuable insights into risk identification and monitoring. When paired with brain imaging techniques like EEG, these methods can effectively aid in diagnosing and assessing cognitive disturbances during SS episodes. Integrated with specially designed computerized cognitive tests or apps, as well as pharmacological/non-pharmacological interventions, these tools could be highly effective for monitoring and managing cognitive disturbances associated with SS.

### Mood and affective symptoms

Mood and affective symptoms such as depression, anxiety, euphoria, irritability, and mood lability form another important cluster of disturbances observed in individuals with dementia, particularly during sundowning periods. These symptoms reflect fluctuations in emotional states and are closely linked to mental health conditions. Although depression and anxiety have been extensively studied, other symptoms like euphoria, irritability, and mood lability have received comparatively less attention. With the advancement of affective computing, multimodal solutions are increasingly being developed to support the detection, diagnosis, and prediction of mood-related disorders. In this study, we aim to utilize multimodal sensors and Emotion AI techniques to capture the nuanced spectrum of mood and emotional changes, offering a more holistic and consistent approach to monitoring affective symptoms.

To effectively identify the risk of mood and affective disorders, multimodal methods can integrate various data sources such as neuroimaging, text, wearable technology, and ambient sensors. For instance, neuroimaging data combined with ML techniques has been used to predict vulnerability to depression (Sato et al., 2015). Text and sentiment analysis using natural language processing also provides valuable insights by analyzing unstructured data from counseling transcripts (Oseguera et al., 2017) and social media (Guntuku et al., 2017) to detect signs of depressive symptoms or suicidal ideation. Additionally, wearable sensors that continuously monitor physiological states and ambient sensors embedded in natural environments can provide real-time data on an individual’s mental health, identifying psychiatric emergencies and tracking ongoing risk factors (Alam et al., 2016; Sano et al., 2015).

The diagnosis of mood and affective symptoms can be significantly enhanced through multimodal methods that integrate psychological or neuroimaging data and video-audio analysis. Neuroimaging techniques, such as EEG and MRI, combined with ML algorithms, have shown promise in predicting diagnoses and distinguishing between mental health conditions with similar symptomatology (Shatte et al., 2019). For example, resting-state MRI scans (Griffith & Hubbard, 2021) and resting EEG functional connectivity (Orgo et al., 2017) have shown potential as diagnostic features for discrimination of depression. By employing ML and deep learning methods in EEG-based depression identification, studies have achieved an overall high classification accuracy of 90% to 100% (for a systematic review, see Dev et al., 2022). Video-audio-based sensors that capture facial expressions, body posture, and both verbal and non-verbal cues via speech and text have also been widely used as diagnostic tools for mental health evaluation, such as depression, and their development has been significantly enhanced by datasets from the Audio/Visual Emotion Challenge and Workshop and the Distress Analysis Interview Corpus Wizard of Oz (Mao et al., 2023). Moreover, extracting remote photoplethysmographic (rPPG) data from video adds a layer of physiological information from video sensors, which could further improve the accuracy of depression detection by offering a richer set of diagnostic markers (Casado et al., 2023; Fan et al., 2024). These additional data enhance the ability to capture subtle physiological changes, providing more comprehensive insights for diagnosing and monitoring mental health conditions.

Monitoring mood and affective symptoms is crucial because they are often subjective, and subtle, and can fluctuate over time. The development of wearable devices, such as smartphones, smartwatches, and fitness bands, equipped with embedded sensors, has made a promising approach to tracking these symptoms. For example, EDA has been identified as a key indicator of depression, suggesting its usefulness in differentiating phases of mood disorders (Sarchiapone et al., 2018). Lower HRV, on the other hand, has been suggested as an important characteristics of both depression (Kemp et al., 2010) and anxiety disorders (Chalfont et al., 2020). Both of these features can be easily measured via various wearable or even contactless sensors (Khan et al., 2024), providing daily life data for monitoring mood and affective symptoms. Moreover, systematic reviews (Rohani et al., 2018) have shown statistically significant correlations between behavioral data (e.g., homestay, screen active duration) collected via mobile and wearable devices in patients with affective disorders, which highlight the growing potential of wearable technologies as powerful multimodal tools for monitoring mood and affective symptoms in real time.

For treatment, multimodal approaches show considerable potential in improving therapeutic outcomes through personalized monitoring and timely feedback. For example, Maseda et al. (2018) found that multisensory stimulation and individualized music sessions had immediate positive effects on mood and behavior in elderly patients with severe dementia, resulting in increased happiness, contentment, and engagement with their surroundings. Physiological improvements, such as decreased heart rate and increased oxygen saturation, were also observed (Maseda et al., 2018). Similarly, a multimodal cognitive enhancement therapy program combining physical and cognitive activities significantly improved mood and cognitive performance in older adults with MCI or mild dementia (Han et al., 2017). Additionally, multimodal approaches integrating physical, mental, and social activities have been associated with improvements in mood and various cognitive functions in healthy older adults, further demonstrating the efficacy of these approaches in both healthy aging and managing mood symptoms (Piccirilli et al., 2019).

In summary, multimodal solutions for detecting and diagnosing mood and affective disorders are becoming increasingly sophisticated by incorporating a range of behavioral and physiological signals. Neuroimaging techniques like EEG and MRI help predict and diagnose conditions such as depression by analyzing brain activity patterns. Wearable sensors, including smartwatches and fitness bands, monitor physiological data like EDA and ECG, both linked to mood fluctuations and depression. Video-audio sensors are used to capture facial expressions and vocal cues, providing insights into mood and emotional states during SS episodes (Xu et al., 2025). Additionally, multimodal approaches that integrate physical, mental, and social activities can enhance mood and cognitive functions in healthy older adults, and assist in managing affective symptoms in SS.

### Psychosis

Psychotic symptoms such as delusions, hallucinations, and illusions have also been observed in individuals with dementia, particularly those experiencing SS (Menegardo et al., 2019; Pyun et al., 2019). These symptoms can increase with the duration and severity of the disease and contribute to institutionalization, cognitive decline, and caregiver burden (Aarsland, 2020). Despite their clinical relevance, psychotic experiences remain difficult to assess due to their subjective and private nature. Current evaluations largely rely on self-reports and clinician interpretations, posing challenges for objective monitoring. This limitation is particularly evident when using conventional sensors, especially in single-modality approaches, highlighting the need for more comprehensive, multimodal sensing strategies to better capture and understand psychotic symptoms in dementia care.

By integrating genetic, neuroimaging, and cognitive data, it becomes more effective to identify risk factors for psychotic symptoms by combining different data types to reveal the complex interplay of factors contributing to psychosis. Neuroimaging data, such as MRI and positron emission tomography (PET), can provide valuable insights into brain abnormalities, whereas genetic data, such as single nucleotide polymorphisms (SNPs), can reveal the risk of developing both psychosis and dementia at earlier stages (Pettersson-Yeo et al., 2013; Venugopalan et al., 2021; Zhou et al., 2019). For example, Pettersson-Yeo et al. (2013) used structural and functional MRI, diffusion tensor imaging (DTI), genetic markers, and cognitive data to successfully differentiate between individuals at ultra-high risk for psychosis, those experiencing their first psychotic episode, and healthy controls at the single-subject level using basic ML techniques like support vector machines (Pettersson-Yeo et al., 2013). Similarly, Schultz et al. (2012) highlighted that multimodal imaging has the potential to shed light on the neuronal mechanisms underlying the major brain structural and functional pathophysiological features of schizophrenia and high-risk states, such as prefrontal-temporal gray matter reduction, altered higher-order cognitive processing, and disturbed dopaminergic and glutamatergic neurotransmission (Schultz et al., 2012).

For diagnosis, though limited, several studies have utilized multimodal imaging techniques to improve the diagnosis of psychosis in dementia patients, focusing on structural and functional brain changes. For instance, single-photon emission computed tomography (SPECT) has been used to detect perfusion patterns in Alzheimer’s patients, showing that psychotic symptoms such as delusions are linked to hypoperfusion in the left frontal lobe, whereas hallucinations are associated with hypoperfusion in the parietal lobe (Kotrla et al., 1995). Also with SPECT scans in Lewy bodies dementia patients, visual hallucinations were related to dysfunction of the parietal and ventral occipital cortices, misidentifications were related to dysfunction of the limbic-paralimbic structures, and delusions were related to dysfunction of the frontal cortices (Nagahama et al., 2010). Furthermore, by investigating the resting-stage EEG source network and the correlation between functional and structural networks from EEG and DTI recordings, Mehraram et al. (2022) found that patients with Lewy body dementia and hallucinations exhibited weakened connectivity within the visual ventral network and between the ventral visual network and the default mode and ventral attention networks. Additionally, the occipital lobe was identified as the most functionally disconnected region, and an association between disruptions in the cholinergic system and functional connectivity abnormalities in patients with hallucinations was identified (Mehraram et al., 2022). Some insights might be also drawn from schizophrenia studies, where AI has been extensively used to assist in diagnosing and predicting schizophrenia through EEG and MRI data across various samples (see, e.g., Yan et al., 2019 for an MRI study and Sun et al., 2021 for an EEG study), which could also be promising for detecting and predicting psychosis in dementia. However, a clearer understanding of the direct effects caused by psychosis symptoms such as inducing these symptoms in healthy subjects could help identify behavioral and neural markers of these symptoms. For episodes of SS-related psychosis symptoms, combining brain imaging methods as biomarkers to confirm the existence of psychosis with video recordings during sunset to monitor and validate the content of sensory stimuli or false beliefs might provide a promising way to offer clinicians valuable assistance in making diagnoses and be considered by future studies.

Multimodal approaches have also demonstrated significant potential in monitoring psychosis symptoms. Smartphone-based solutions that integrate self-reported data, behavioral sensing (e.g., physical activity, geospatial activity, speech data), and smartphone usage patterns offer a flexible and scalable approach to symptom monitoring. One notable example is the CrossCheck system, which has shown promise in identifying digital indicators of psychotic relapse, particularly by analyzing and incorporating personalized indicators from different participants (Ben-Zeev et al., 2017). Similarly, other mobile applications that allow patients to complete self-reported assessments for monitoring ambulatory key psychosis symptoms (Palmier-Claus et al., 2012) or track basic symptoms and early signs of psychosis relapse (Eisner et al., 2019) have also demonstrated the capability to continuously monitor psychosis symptoms, such as delusions and hallucinations, and provide early warnings of relapse or symptom escalation.

Unlike other symptoms that tend to exhibit more general patterns among groups, such as heightened activity levels or expressions of negative emotions, psychotic symptoms are highly subjective and unique to each individual. The frequency, severity, and impact of these symptoms can fluctuate significantly within a person over time, influenced by both external environmental conditions and internal affective states. This variability underscores the importance of systems like CrossCheck (Ben-Zeev et al., 2017), which enables a more comprehensive understanding of these individual symptom fluctuations. By collecting multimodal data, these systems can help clinicians tailor timely interventions based on personal symptom profiles. However, it is regrettable that multimodal approaches specifically designed for the treatment of psychosis, particularly in patients with dementia or SS, remain under-researched. This may be due to the complexities of studying psychosis itself, as well as the challenges in developing comprehensive monitoring systems. Nonetheless, as mentioned previously, the integration of multimodal data, coupled with the creation of personalized profiles, holds great potential for future research and treatment.

In sum, neuroimaging techniques, including MRI, PET, and EEG, could be used to assess brain abnormalities and connectivity issues linked to psychosis, providing valuable insights into the neurological underpinnings of these symptoms. Genetic data, such as SNPs, can help identify risk factors for developing psychosis in dementia patients. Video recordings combined with brain imaging during sunset can help validate sensory stimuli or false beliefs experienced during SS episodes. Additionally, smartphone-based systems like CrossCheck tracks behavioral patterns, speech data, and physical activity to monitor psychotic symptom fluctuations and offer early warnings of relapse. In turn, with advancements in technology and personalized medicine, multimodal approaches could become a highly promising direction for managing psychotic symptoms in patients with SS, enabling more precise and effective care tailored to individuals.

### Changes in activities of daily living and changes in instrumental activities of daily living

SS can significantly affect both ADLs and IADLs, often resulting in changes that do not fall neatly into the previously discussed symptom categories. These include dietary changes (Dechamps et al., 2008), sleep disturbances (Satlin et al., 1992), and behavioral issues such as resistant behavior, hoarding, or repetitive searching and cleaning (Sevilla et al., 2018), which may be closely tied to disrupted evening routines. Understanding these changes within the broader framework of ADL and IADL functioning is essential for comprehensive SS management. Unlike more well-defined clinical symptoms, these manifestations often reflect broader behavioral patterns and lifestyle disruptions. Therefore, our focus lies in exploring effective monitoring and intervention strategies using multimodal tools, with an emphasis on early risk detection and personalized support rather than rigid symptom classification.

The Internet of Things (IoT) represents a technological revolution in which virtually anything can be connected and communicate intelligently (Madakam et al., 2015). By its nature, IoT integrates various types of sensors, such as wearable, environmental, and physiological sensors, that work together to enable chronic disease management, early diagnosis, real-time monitoring, and emergency response in a cost-effective way. This integration could enhance interactions among individual patients, clinics, and healthcare organizations in the context of healthcare (Islam et al., 2015). Utilizing data mining and ML techniques from wearable or wireless sensors around the home, it is possible to monitor most ADLs such as bathing, dressing, toileting, eating, and sleeping, as well as some IADLs like using the telephone, preparing meals, and managing medications (Pol et al., 2013). This technology enables the design of algorithms to detect uncommon patterns, such as those occurring during sundowning time, providing timely interventions or alerts. For example, Enshaeifar et al. (2018) used home-installed IoT sensors, such as passive infrared sensors, pressure sensors, door sensors, and physiological monitoring sensors for heart rate, blood pressure, and body temperature, to profile and analyze patients’ daily routines and detect unusual patterns and agitation, irritation, and aggression events with an accuracy of up to 80% (Enshaeifar et al., 2018).

Another potential trend is the use of mobile apps for various ADL/IADL management. Currently, many apps have been developed to provide ADL-based cognitive training, monitoring, dementia screening, reminiscence, socialization, tracking, and caregiver support for the healthcare of dementia (for a comprehensive review, see Yousaf et al., 2020). For instance, the Dementia/Digital Diary/Clock app (2015) displays both digital and analog clocks and provides a remotely configured calendar, which helps PWD feel connected with family members, reducing confusion and enhancing control in daily living (Yousaf et al., 2020). In another case, Burleson et al. (2018) developed and evaluated a prototype system that includes an app to connect PWD and caregivers, along with various sensors such as cameras and motion detectors placed on top of a dresser. This system guides PWD through the dressing process, addressing typical issues like donning a shirt or pants inside out or backward, and partial dressing. Despite some misidentifications, this system offers potential automated dressing support to assist PWD, a challenging task for both individuals with dementia and their caregivers (Burleson et al., 2018).

Although there are currently no IoT systems or apps specifically developed for SS, we believe that with the advancement of these technologies, detailed recording of a patient’s ADL and IADL to form personalized profiles and provide more individualized detection and timely assistance will become feasible. As different sensors develop and smart home and mobile devices become more prevalent, these functionalities for tracking patients’ daily activities and recording specific environmental information such as time and location are expected to become valuable tools for documenting and intervening in SS in the future.

**Fig. 3:**
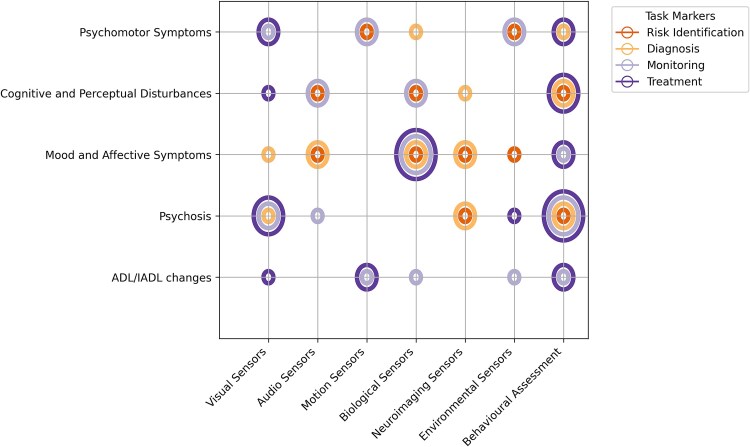
Symptoms and sensor types represented with task markers. The tasks are categorized as follows: risk identification, diagnosis, monitoring, and treatment. The figure demonstrates how various multimodal sensors, such as visual, audio, motion, biological, neuroimaging, environmental, and behavioral assessments, can be combined for different tasks related to specific symptom categories. The size of the circles reflects the involvement and overlap of sensors but does not carry any specific quantitative significance.

In this section, we discussed various symptoms and corresponding multimodal solutions used to assess them, particularly in different situations and applications. Table 3 summarizes the core application strategy for each symptom category, as well as the measurable metrics and their corresponding sensors mentioned earlier. Building on this, Fig. 3 provides a simplified visual mapping of various subtasks associated with different sensor types discussed in this study. This figure demonstrates how different sensors, including visual sensors, audio sensors, motion sensors, biological sensors, neuroimaging sensors, environmental sensors, and behavioral assessments, can be integrated within a multimodal recording platform to capture diverse features for different symptom categories under SS. The aim is to provide a clearer perspective on how these sensors can be combined to assess symptoms across various scenarios. Please note that this framework serves only as an initial guide to understanding the multi-task associations identified in this study and is based on prior research. Further studies are needed to comprehensively explore and refine these sensor-symptom mappings.

**Table 3:** Example solutions for risk identification, diagnosis, monitoring, and treatment of various symptoms associated with sundown syndrome

**Category**	**Risk Identification**	**Diagnosis**	**Monitoring**	**Treatment/Response**
**Psychomotor symptoms**	– **Objective:** Early identification of behavioral indicators under stressful conditions	– **Objective:** Detection of specific motor symptoms indicative of agitation or pacing through multimodal data analysis	– **Objective:** Continuous monitoring of activity patterns to identify behavioral indicators of increased aggression, wandering, or restlessness	– **Objective:** Assessment of therapeutic interventions and real-time strategies for improving psychomotor control
	– **Metrics:** Activity levels, environmental stimuli, movement patterns	– **Metrics:** Agitation events (motor, verbal, physical), physiological and neural activity	– **Metrics:** Motor activity, walking speed, location, movement irregularities	– **Metrics:** Motor coordination, physical activity, response to therapy
	– **Sensors:** Motion sensors (actigraphy), environmental sensors (PIR)	– **Sensors:** Biometric sensors (Empatica E4), visual sensors (video), neuroimaging (EEG)	– **Sensors:** Motion sensors (actigraphy, GPS), visual sensors (video), environmental sensors (PIR)	– **Sensors:** Biometric sensors, VR, digital interventions
**Cognitive disturbances**	– **Objective:** Early detection of cognitive decline prior to confusion or delirium	– **Objective:** Confirmation of cognitive impairments through cognitive assessments and multimodal sensor integration	– **Objective:** Longitudinal tracking of cognitive status to identify confusion or disorientation related to time, place, or person	– **Objective:** Assessing treatment effects on cognition and proposing integrated solutions for improved cognitive function
	– **Metrics:** Performance in cognitive tasks, speech, physiological and language processing changes	– **Metrics:** Cognitive performance and neural activity	– **Metrics:** Linguistic analysis, cognitive ability, physiological state	– **Metrics:** Improvement in cognitive performance, communication ability
	– **Sensors:** Biometric sensors (ECG, eye tracking), audio sensors, electronic health records	– **Sensors:** Neuroimaging (EEG, MRI), cognitive tests (MMSE, MoCA)	– **Sensors:** Audio sensors, biometric sensors, mobile app, neuroimaging (EEG)	– **Sensors:** Digital apps
**Mood and affective symptoms**	– **Objective:** Early identification of mood disturbances, such as dysphoria or restlessness, which may predict emotional dysregulation	– **Objective:** Diagnosing affective disorders based on combined behavioral and physiological markers	– **Objective:** Longitudinal monitoring of emotional fluctuations to assess symptoms of depression, anxiety, and other mood disorders	– **Objective:** Evaluating treatments for affective symptoms and applying combined therapeutic approaches to improve emotional stability
	– **Metrics:** Interpersonal communication, emotional response, physiological changes	– **Metrics:** Neural activity indicators, speech, text, and facial/bodily expression analysis, rPPG	– **Metrics:** Mood tracking, physiological response, self-reports	– **Metrics:** Emotional regulation, mood improvement
	– **Sensors:** Audio sensors, biometric sensors, environmental sensors	– **Sensors:** Visual sensors, Neuroimaging (EEG, MRI), video-audio sensors	– **Sensors:** Biometric sensors (EDA, ECG), mobile apps	– **Sensors:** Multisensory stimulation, biometric sensors (HR, SpO_2_)
**Psychosis**	– **Objective:** Early identification of warning signs, such as subtle changes in thought patterns, perception, or behavior	– **Objective:** Confirmation of psychotic symptoms through personalized assessments and multimodal data integration	– **Objective:** Continuous monitoring of psychotic episodes to assess frequency and severity of hallucinations and delusions	– **Objective:** Measurement of antipsychotic efficacy and the introduction of new therapeutic combinations for symptom reduction
	– **Metrics:** Genetic information, neuroimaging markers	– **Metrics:** Brain structural and functional features, contextual information	– **Metrics:** Self-reports, personalized indicators, smartphone usage patterns	– **Metrics:** Individual symptom fluctuations, personal symptom profiles
	– **Sensors:** Neuroimaging (EEG, MRI, DTI, PET), genetic testing	– **Sensors:** Neuroimaging (MRI, EEG, SPECT), visual sensors	– **Sensors:** Biometric sensors, visual sensors, motion sensors, mobile app	– **Sensors:** Biometric sensors, visual sensors, motion sensors, mobile app
**ADL/IADL**	– Not applicable	– Not applicable	– **Objective:** Tracking of daily activities (e.g., sleep, appetite) to detect disruptions in self-care routines	– **Objective:** Evaluation of treatments for enhancing daily functioning and development of new strategies for improved outcomes
			– **Metrics:** IoT, daily routine tracking, movement monitoring	– **Metrics:** IoT, daily activity improvements, ADL-based cognitive training
			– **Sensors:** Environmental sensors (PIR, pressure sensors, door sensors, etc.), biometric sensors	– **Sensors:** Visual sensors, motion sensors, mobile app

*Notes:* Objective represents the core application strategy for each symptom category, following the BEST framework (see Box 1). Metrics refer to the key cognitive, emotional, or behavioral features being monitored. Sensors denote the devices used to collect the corresponding data. Sensors listed in parentheses highlight modalities or specific sensors that have been validated and referenced in this review. PIR = passive infrared sensors; EEG = electroencephalography; MRI = magnetic resonance imaging; ECG = electrocardiogram; EDA = electrodermal activity; rPPG = remote photoplethysmography; SpO_2_ = oxygen saturation; PET = positron emission tomography; SPECT = single-photon emission computed tomography; GPS = Global Positioning System; IoT = Internet of Things

## CONCLUDING REMARKS

This review has systematically synthesized previous research on SS, focusing on its key symptom domains, measurement strategies, and technological advancements. It presents a comprehensive and operational framework to support the development of multimodal solutions aimed at improving risk identification, diagnosis, monitoring, and treatment. By categorizing common symptoms and suggesting targeted sensor-based measurements, we lay the groundwork for future clinical and technological applications. To translate these efforts into a practical strategy, we propose a structured five-step roadmap for multimodal SS monitoring that bridges the gap between symptom clustering and real-world implementation.

- **Step 1: Symptom classification** 
  Building on existing literature and our synthesis of common SS-related manifestations, we categorize SS symptoms into five primary domains: (1) psychomotor symptoms, (2) mood and affective symptoms, (3) psychosis, (4) cognitive and perceptual disturbances, and (5) changes in ADLs and IADLs. This classification provides the foundational structure for aligning symptoms with specific measurement strategies.
- **Step 2: Feature identification** 
  For each symptom category, key features are identified that can be quantitatively or qualitatively captured through sensors. For instance, agitation can be reflected through elevated physical activity or frequent location transitions. Cognitive disturbances may be associated with slower speech rate, impaired eye tracking, or reduced HRV. These features offer clear and measurable targets for selecting appropriate sensors.
- **Step 3: Sensor mapping** 
  Each feature is matched with appropriate sensing modalities, including wearable sensors (e.g., actigraphy, ECG, EDA), ambient sensors (e.g., passive infrared sensors, smart lighting, door contacts), audio-video sensors (e.g., microphones, cameras, facial and vocal emotion detection), neuroimaging (e.g., EEG, MRI), and mobile-based behavioral data (e.g., app interactions, digital biomarkers). These sensors form the technological backbone for multimodal monitoring.
- **Step 4: Temporal and symptom integration** 
  Given SS’s characteristic late-day symptom exacerbation, it is critical to synchronize symptom-specific data streams across domains and align them with time-stamped inputs. This approach helps identify clinically meaningful temporal patterns characteristic of SS and supports a unified view of complex symptom interactions.
- **Step 5: Actionable outcome generation** 
  Multimodal data are synthesized to generate actionable outcomes that can be directly used by healthcare professionals, structured around the four clinical phases emphasized in this review: (1) risk identification through early behavioral alerts; (2) diagnosis via sensor-informed symptom profiling; (3) monitoring of daily fluctuations using continuous data; and (4) treatment response evaluation through real-time feedback and personalized intervention support.

**Fig. 4:**
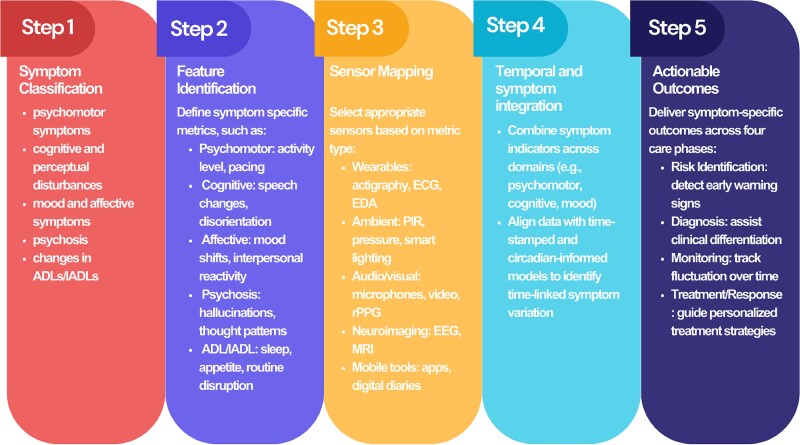
Five-step roadmap for multimodal monitoring of sundown syndrome. Feature examples and sensor types in Steps 2 and 3 reflect mappings from Table 3, which links symptom domains with measurable metrics and corresponding sensor technologies. Step 5 incorporates the BEST framework emphasized in this review, aiming to deliver actionable, timely, and caregiver-oriented solutions for real-world implementation.

This five-step roadmap is visually illustrated in Fig. 4 and complements the sensor-task integration strategies presented in Table 3 and Fig. 3. While Table 3 provides detailed mappings between symptom categories, measurable features, sensor types, and actionable outcomes, Fig. 3 offers a broader view of how different sensor modalities can be applied across tasks and symptom domains. Figure 4 further brings these elements together into a coherent, process-oriented framework. Together, these visual elements enhance the conceptual and operational clarity of the proposed approach. By implementing this roadmap, we offer a structured, context-aware blueprint for integrating multimodal sensing and time-sensitive analytics into SS research and care practices, ultimately facilitating earlier detection, more accurate diagnosis, and personalized intervention strategies. However, despite the progress outlined in this review, several limitations and future directions still need to be addressed:

- **Data limitations:** One of the main difficulties is the lack of reliable and relatively large-scale data. Research on SS is still fragmented, with many studies facing replication challenges due to a lack of detailed clinical feature descriptions and objective sensor recording. Therefore, gathering comprehensive, high-quality datasets will be essential for advancing SS research and for refining the measurement and treatment methodologies.
- **Technological barriers:** In addition to data limitations, technology presents another challenge. Although advanced big data processing tools and large foundation models offer the potential for improving SS measurement, they have yet to be fully utilized in SS research. Specifically, the reliability of the proposed signals in detecting specific conditions in SS remains uncertain, especially when it comes to distinguishing between different symptoms that share similar features within each symptom category. Future work should focus on developing robust systems for handling and analyzing large-scale, multimodal datasets, which will be critical for enhancing the accuracy of SS identification and treatment recommendations.
- **Neglect of environmental factors:** Although some studies have explored environmental influences, previous research has primarily focused on patient-centered factors, with limited attention given to the environment itself. For instance, higher light levels have been shown to enhance alertness and cognitive performance by affecting the hypothalamus (Campbell et al., 2024). Recently, research has increasingly highlighted the role of environmental factors, particularly light exposure, in managing SS (Guu et al., 2022). Integrating measurements of environmental conditions with patient data may support more personalized and natural interventions. To address this, future research should explore the use of ambient sensors to monitor and adjust light exposure within a multimodal framework, potentially incorporating smart lighting systems (Zeng et al., 2022) to help manage sleep disturbances and SS symptoms.
- **Animal models and cross-species research:** Furthermore, current research predominantly focuses on human studies, and although modeling the circadian and affective components of SS in mice is challenging, some studies related to AD pathology have identified potential models that could be applied to SS (for a review, see Bedrosian & Nelson, 2013). For example, 6-month-old transgenic APP23 mice have been shown to exhibit altered activity profiles toward the end of their active period, which closely resembles SS behavior in dementia patients. This bimodal nocturnal activity pattern becomes even more pronounced by 12 months of age, suggesting that transgenic APP23 mice could serve as a potential model for SS (Vloeberghs et al., 2004). Similarly, a more recent study found that APPSwe-Tau (TAPP) mice demonstrated a phase delay in body temperature and locomotor activity, linking circadian dysfunction and increased aggression to Tau pathology in the lateral parabrachial nucleus, thus providing a temporal and circuit-based foundation for SS (Warfield et al., 2023). Additionally, although limited, similar behavioral changes have also been observed in aging dogs and cats with cognitive dysfunction (e.g., Černá et al., 2020; Piotti et al., 2022). Future research should continue to investigate these mechanisms and incorporate cross-species studies to identify relevant biomarkers. Nonetheless, differences in natural circadian rhythms between species, such as the reversal of these rhythms in mice compared to humans, must be carefully considered.
- **Ethical considerations:** Finally, ethical concerns, especially regarding patient privacy and the trustworthiness of AI systems, must be prioritized as technologies are increasingly applied to the elderly population, including SS patients. Ensuring adherence to general guidelines, such as requiring human supervision and monitoring and compliance with applicable privacy laws and regulations, as outlined in ethical codes by previous studies (Koocher & Keith-Spiegel, 2008; Luxton, 2014), is essential. Following these ethical standards will contribute to a more holistic approach to SS research and care.

## Supplementary Material

Supplementary_Table1_acaf062

Supplementary_Table2_acaf062

## Figures and Tables

**Appendix A:** Abbreviation list

**Abbreviation**	**Full Term**	**Abbreviation**	**Full Term**
Aβ42	Beta-amyloid	ML	Machine learning
ABMI	Agitation Behavior Mapping Instrument	MMSE	Mini-Mental State Examination
ABRS	Agitated Behavior Rating Scale	MoCA	Montreal Cognitive Assessment
ADLs	Activities of daily living	MRI	Magnetic resonance imaging
AI	Artificial intelligence	NEECHAM	Neelon and Champagne Confusion Tool
APOEɛ4	Apolipoprotein E4	NPI	Neuropsychiatric inventory
AR	Augmented reality	NPS	Neuropsychiatric symptoms
AUC	Area under the curve	PET	Positron emission tomography
BEST framework	Biomarkers, Endpoints, and other Tools framework	PIR	Passive infrared
CAM	Confusion Assessment Method	pTau	Phosphorylated tau
CI	Confusion Inventory	rPPG	Remote photoplethysmographic
CMAI	Cohen–Mansfield Agitation Inventory	SBF	Sundowning behavior flowsheet
CSF	Cerebrospinal fluid	SEQ	Sundowning Evaluation Questionnaire
ECG	Electrocardiogram	SNPs	Single nucleotide polymorphisms
EDA	Electrodermal activity	SPECT	Single-photon emission computed tomography
EEG	Electroencephalography	SS	Sundown syndrome
FLL	Full listening log	SSQ	Sundown Syndrome Questionnaire
fMRI	Functional magnetic resonance imaging	TBDQ	Time-Based Behavioral Disturbance Questionnaire
HRV	Heart rate variability	VR	Virtual reality
IADLs	Instrumental activities of daily living		
